# Application of PZT Ceramic Sensors for Composite Structure Monitoring Using Harmonic Excitation Signals and Bayesian Classification Approach

**DOI:** 10.3390/ma14195468

**Published:** 2021-09-22

**Authors:** Michal Dziendzikowski, Mateusz Heesch, Jakub Gorski, Krzysztof Dragan, Ziemowit Dworakowski

**Affiliations:** 1 Airworthiness Division, Air Force Institute of Technology, 01-494 Warszawa, Poland; krzysztof.dragan@itwl.pl; 2 Department of Robotics and Mechatronics, Faculty of Mechanical Engineering and Robotics, AGH University of Science and Technology, 30-059 Krakow, Poland; heesch@agh.edu.pl (M.H.); jgorski@agh.edu.pl (J.G.); zdw@agh.edu.pl (Z.D.)

**Keywords:** Structural Health Monitoring, impact damage detection, composite structure monitoring, Bayesian classification, PZT transducers applications

## Abstract

The capabilities of ceramic PZT transducers, allowing for elastic wave excitation in a broad frequency spectrum, made them particularly suitable for the Structural Health Monitoring field. In this paper, the approach to detecting impact damage in composite structures based on harmonic excitation of PZT sensor in the so-called pitch–catch PZT network setup is studied. In particular, the repeatability of damage indication for similar configuration of two independent PZT networks is analyzed, and the possibility of damage indication for different localization of sensing paths between pairs of PZT sensors with respect to damage locations is investigated. The approach allowed for differentiation between paths sensitive to the transmission mode of elastic wave interaction and sensitive reflection mode. In addition, a new universal Bayesian approach to SHM data classification is provided in the paper. The defined Bayesian classifier is based on asymptotic properties of Maximum Likelihood estimators and Principal Component Analysis for orthogonal data transformation. Properties of the defined algorithm are compared to the standard nearest-neighbor classifier based on the acquired experimental data. It was shown in the paper that the proposed approach is characterized by lower false-positive indications in comparison with the nearest-neighbor algorithm.

## 1. Introduction

Structural Health Monitoring (SHM) is a key technology for further advancement and evolution of industry, transport, civil engineering, or space exploration paradigms. Increased automation and optimization of industrial processes, widespread use of autonomous Unmanned Aerial Vehicles (UAVs), or design of constructions subjected to extreme load or environmental conditions will require developing technology for continuous structural integrity monitoring of critical components and early damage detection systems. Among various approaches to SHM [1,2,3,4,5] PZT ceramic sensors have proven to be the technology of the universal application capabilities [6,7,8,9]. Application of PZT transducers provides a possibility to excite and receive elastic waves in all kinds of continuous media [6,10]. Elastic waves can interact with structure discontinuities caused by different kinds of damage enabling successful PZT use for Structural Health Monitoring. There exists a vast literature on the subject, providing numerous examples of PZT application [11], in particular to:

- cracks detection and their growth monitoring [12,13,14];
- bolt and rivet joints monitoring [15,16,17,18,19];
- corrosion detection [20,21,22];
- concrete structures and soil properties monitoring [23,24,25,26];
- pipeline damage detection [27,28];
- large structure monitoring [29,30,31,32];
- malignant tumor detection [33];
- other applications [34,35].

**Figure 1:**
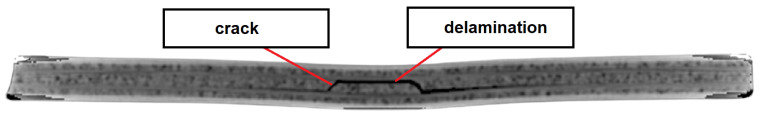
Cross section visualization of an impact damage of composite structure obtained with use of computer tomography.

In addition to the PZT applications mentioned above, monitoring of composite structures is of the utmost importance, especially in the aerospace industry [36,37]. Composites, due to their high durability and lightweight are commonly used for civil [37,38] and military [39,40] aircraft manufacturing. Their application allowed to increase performance and reduce fuel consumption of modern aircraft. Despite the many advantages of composite structures, there are also some drawbacks of their usage. Unlike metal alloys, fatigue properties of composite structures and methods of their remaining fatigue durability under damage presence are yet to be fully developed. Those parameters can depend not only on the properties of constituent layers but also on their layup. Therefore, significantly more data are needed for fatigue composite structure characterization than in the case of metallic structures [41]. Additionally, composites are vulnerable even to low-energy impacts, which can introduce in the structure the so-called Barely Visible Impact Damage (BVID) [42]. Impacts can introduce transverse cracks of layers and multiple subsurface delamination in the material (Figure 1), resulting in decreased stiffness and durability of the structure [43,44,45]. BVID can be barely visible on the surface of the material. Therefore, advanced non-destructive methods need to be applied for damage evaluation, e.g., ultrasonic testing (UT) [45,46].

Significant development of SHM system with the use of PZT transducers for composite structure monitoring was performed [8,9,37,47], in particular towards BVID detection and monitoring [48,49,50,51].

In this paper, a method for BVID detection and classification based on PZT transducers application is presented. The steady-state harmonic voltage signal is used for guided wave excitation in the pitch-catch PZT network configuration. This setup is rarely used compared to classic approaches such as the Electromechanic Impedance technique or pitch-catch pulsed excitation of guided waves [7,8]. Signal evaluation is based on Damage Indices maintaining complete information on the phase and the amplitude of the response signals, following definition introduced in [52]. In [52], the properties of the approach were examined with respect to artificially introduced damage. In this paper, it is applied to BVID damage detection of composites structures caused by low-energy impacts. Another novelty of this work lies in the definition of a new Bayesian approach to SHM data classification. The Bayesian classifier is based on asymptotic properties of Maximum Likelihood estimators [53] and Principal Component Analysis for orthogonal data transformation [54]. Both the complete Bayesian classification algorithm and its simplified version, proper for direct implementation in the Python environment, are provided in the paper. Properties of the defined algorithm are compared to the well-known nearest-neighbor classifier [54] based on experimental data.

The paper is organized as follows. In the first two sections, methods used for data acquisition and classification are presented, i.e., in Section 2, the approach to damage detection with the use of PZT sensors is introduced, and in Section 3 data classification model based on Bayesian paradigm is defined. Section 4 provides experimental verification of the proposed methods. In particular, repeatability of damage indication for similar configuration of two independent networks of sensors is presented. As discussed in [13,14], this property is of particular importance in real applications since it allows the training of an appropriate model for BVID detection on a model PZT network and use it for the classification of data acquired for other PZT networks installed on target structures. Additionally, the efficiency of the defined Bayesian classifier is evaluated and compared to results obtained with the use of the nearest-neighbor classifier. In Section 5, the paper is concluded.

## 2. Structural Health Monitoring Based on Harmonic Excitation of PZT Sensors and Voltage Transfer Ratio

For SHM systems based on elastic wave excitation by a network of PZT transducers, there are two factors that define in general a given approach to structure monitoring [3,7,8,9]:

- the configuration of PZT transducers;
- the type of excitation voltage applied to PZT actuators.

Considering network configuration, a single PZT transducer in the network can be used as a guided waves generator or receiver. Both functions can be adopted simultaneously. In the so-called pulse-echo setup, a single PZT transducer can be used for damage detection. In that case, the idea for structure monitoring is based on the acquisition of elastic waves emerging from PZT transducer which are scattered and reflected from structure discontinuities surrounding it. The amplitude of the received signal depends on wave reflection coefficient on damage for a given excitation frequency [55], the distance of damage from the PZT sensor as well as attenuation properties of the monitored structure. This may, in some cases, restrict the range of sensor efficiency, in particular when excitation voltage applied to PZT transducer is relatively small [11]. Additionally, pairs of PZT transducers can be used for structure monitoring. Then one of the transducers is used for elastic wave excitation and the other as a receiver. This approach is called pitch-catch setup. Application of this setup to SHM provides more flexibility of signal analysis since depending on the relative localization and orientation of sensing path and damage, both transmission and reflection modes of elastic waves interaction with damage can be used for structure assessment. In addition, there are also two approaches to guided wave excitation: PZT actuator can be sourced with a short-pulsed or steady-state sinusoidal voltage. In practice, the most commonly used approach is to apply pulsed excitation in the pitch-catch transducers scheme [8,9] or to use steady-state harmonic voltage source in the pulse-echo setup as in electromechanical impedance (EMI) technique [7,24,56,57].

**Figure 2:**
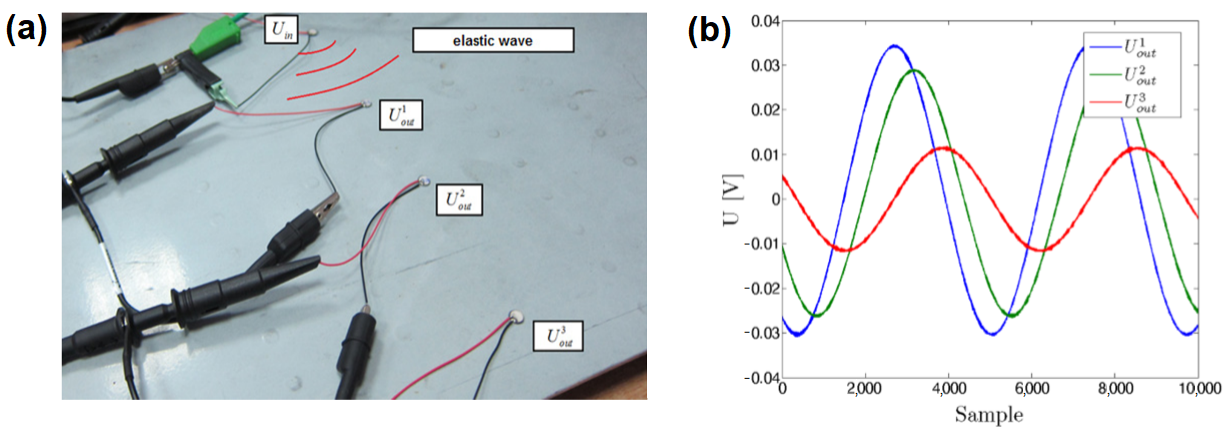
Example of a sinusoidal steady–state excitation of PZT sensors [52]: (**a**) PZT sensors configuration; (**b**) output voltages acquired on PZT sensors.

In this study, a pitch-catch PZT network setup with harmonic voltage excitation is used for BVID detection. This approach is also used for SHM purposes [29,52,58,59,60], although not to the same extent as EMI or standard pitch-catch techniques [61]. For signal analysis, the approach presented in [52] will be followed. In this approach, the input voltage $U_{in}$ applied to PZT transducer used as elastic waves actuator is sinusoidal signal with angular frequency ω similarly as for EMI technique. The response signal used for structure assessment in that case is voltage $U_{out}$ induced on PZT sensor used as elastic waves receiver. In the linear case, which should hold for sufficiently low excitation signal and in appropriate frequency range not containing resonant frequency of PZT transducers, the output transducer voltage $U_{out}$ is also sinusoidal with the same frequency as $U_{in}$ but can have different amplitude and it can be phase-shifted with respect to $U_{in}$. In Figure 2b, an example of voltage signals obtained for three different PZT sensors (Figure 2a) receiving elastic waves excited by an actuator sourced with sinusoidal voltage is presented. The three sinusoidal signals differ in the amplitude and the phase between them, which depend on their location with respect to PZT actuator excited with sinusoidal voltage $U_{in}$.

In that case the ratio between output and input voltage, called forward voltage transfer ratio (FVTR):

(1) $$FVTR=\frac{U_{out}}{U_{in}}$$

can be written in complex form as:

(2) $$FVTR\left(\omega \right)=\frac{U_{out}}{U_{in}}=\frac{|U_{out}|e^{i(\omega t+\varphi (\omega ))}}{|U_{in}|e^{i\omega t}}=\left|FVTR\left(\omega \right)\right|e^{i\varphi (\omega )}$$

where |FVTR(ω)| and φ(ω) denote respectively—the amplitude ratio and the phase difference between output $U_{out}$ and input $U_{in}$ signals at a given frequency. Both components of FVTR can contain information about the eventual damage of the structure within the sensing range of a given sensing path. In principle, it is very hard to find indication of eventual damage based only on FVTR function acquired for unknown state of the structure, since it can be dependent on all mechanical properties of the structure within the sensing range of a given sensing path, whether damage dependent or not. Therefore, damage detection and structure assessment are usually performed using signal characteristics, called the Damage Indices (DIs), which are based on a comparison of the baseline signal—acquired for the pristine state of the structure with the signal obtained for the actual structure condition and are designed to capture changes of signal eventually caused by damage. Denoting as FVTR(ω) the voltage transfer ratio obtained for the actual state of the structure and as $FVTR_{0}\left(\omega \right)$ the baseline voltage transfer ratio, the DIs useful for structure assessment can be defined as the ratio of the two transfer functions:

(3) $$DI\left(\omega \right)=\frac{FVTR(\omega )}{FVTR_{0}\left(\omega \right)}=\frac{\left|FVTR(\omega )\right|}{|FVTR_{0}\left(\omega \right)|}e^{i(\varphi \left(\omega \right)-\varphi_{0}\left(\omega \right))}.$$

**Figure 3:**
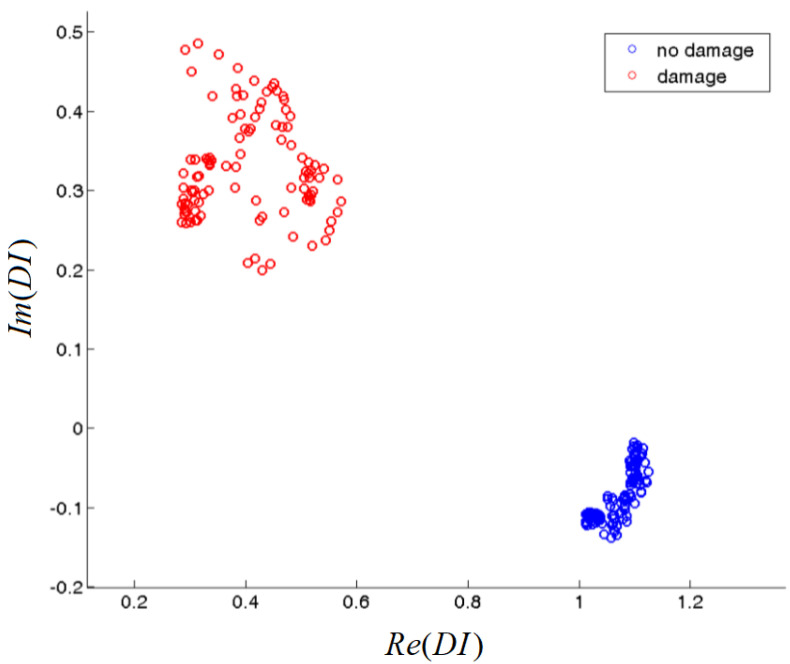
Example of the DIs obtained for the pristine state of the structure and when a damage is present [52].

The above Damage Index is defined for a given excitation frequency. However, DIs obtained for a range of frequencies can be used for damage detection and classification. It is worth noticing that no information is lost, DIs capture all the information about the output voltage amplitude and its phase changes, assuming that the baseline $FVTR_{0}$ is known. Figure 3 illustrates an example of DIs behavior obtained for a range of frequencies for undamaged structure and in the presence of damage [52]. For undamaged state, the DIs are concentrated in the vicinity of the point 1+i0 in the complex plane, irrespectively of the excitation frequency. If damage is present, it can change the output voltage amplitude and its phase. Therefore, DIs diverge from the point 1+i0, at least for frequencies sensitive to damage. A necessary condition for SHM methods to work correctly is the repeatability of damage indications under similar conditions. To achieve that, DIs should be located at the same region of the complex plane when comparable damage is present near a sensing path. This property was demonstrated for particular type of damage in [52] and will also be discussed in this paper.

## 3. Definition of Data Classification Method

For automated structure assessment, having defined signal analysis methods, including Damage Indices allowing for distinction of signal differences due to damage, in addition also methods of data classification are needed. There are many examples of classical data classification approaches, e.g., linear/quadratic discriminant analysis, nearest-neighbor method, Support Vector Machines or neural networks models [54] which can be applied to SHM. In the paper a framework for data classification based on Bayesian inference is proposed. Bayesian approach, due to its generality and flexibility, is often adopted for data classification in the SHM field [62,63,64,65,66].

### 3.1. General Bayesian Setup

A general definition of Bayesian model in the SHM context can be formulated as follows. Assuming there exists a finite set of predefined structure conditions $M_{1},\ldots ,M_{k}$, the probability distribution of continuous Damage Indices $\left(DI_{1},\ldots ,DI_{m}\right)$ adopted for structure assessment denoted as $p(DI_{1},\ldots ,DI_{m}|M_{j})$, can depend on the state of the structure $M_{j}$ within the sensing range of a given sensing path. In the framework it is assumed that state of the structure can be described by a categorical variable. It is true if SHM system is required only to indicate damage presence in the structure or distinguish between different types or stages of developing damage, therefore if the structure needs to be assessed in continuous terms, e.g., by precise location or size of damage, the framework needs to be modified accordingly. Although the paper is focused on application of the voltage transfer ratio to SHM, for the purpose of definition of Bayesian framework for data classification the notation will be kept general, since it can be also used for other approaches as well. In Section 3.4 the details of classifier setup used in this particular paper are provided.

Based on the set of Damage Indices $\left(DI_{1},\ldots ,DI_{m}\right)$ obtained for unknown state of the structure, Bayesian classification of the observed data is based on the so-called a posteriori probabilities obtained from Bayes formula [67]:

(4) $$p\left(M_{j}|DI_{1},\ldots ,DI_{m}\right)=\frac{p\left(DI_{1},\ldots ,DI_{m}|M_{j}\right)w_{j}}{p(DI_{1},\ldots ,DI_{m})}$$

where $p(M_{j}|DI_{1},\ldots ,DI_{m})$ denotes probability of structural state occurrence given measurement output $\left(DI_{1},\ldots ,DI_{m}\right)$, $w_{j}$ is set of weights describing a priori probabilities of structural state $M_{j}$ occurrence and $p(DI_{1},\ldots ,DI_{m})$ is total probability density of the obtained measurement output $\left(DI_{1},\ldots ,DI_{m}\right)$:

$$p\left(DI_{1},\ldots ,DI_{m}\right)=\sum_{j}p\left(DI_{1},\ldots ,DI_{m}|M_{j}\right)w_{j}.$$

One of the most common Bayesian classifiers maximize a posteriori probability or equivalently a posteriori factors $PF_{j}$, given by:

(5) $$PF_{j}=p\left(DI_{1},\ldots ,DI_{m}|M_{j}\right)w_{j}.$$

The probability densities $p(DI_{1},\ldots ,DI_{m}|M_{j})$ governing measurements output in terms of Damage Indices under given structural state are not always specified as a single distribution. Instead, when it is hard to precisely define parameters of the distribution, $p(DI_{1},\ldots ,DI_{m}|M_{j})$ can be defined as integral over selected parameter family of probability densities:

(6) $$p\left(DI_{1},\ldots ,DI_{m}|M_{j}\right)=\int_{\Theta_{j}}p_{\left(\theta_{1},\ldots ,\theta_{n}\right)}\left(DI_{1},\ldots ,DI_{m}|M_{j}\right)\pi_{j}\left(\theta_{1},\ldots ,\theta_{n}\right)d\theta_{1}\ldots d\theta_{n},$$

where $p_{\left(\theta_{1},\ldots ,\theta_{n}\right)}$ is parametric distribution family on the space spanned by the defined Damage Indices $DI_{1},\ldots ,DI_{m}$ with unknown parameters $\left(\theta_{1},\ldots ,\theta_{n}\right)\in \Theta_{j}\subset R^{n}$ and $\pi_{j}$ is a probability density on the allowable parameter space $\Theta_{j}$. As an example, if a single Damage Index DI would be used for structure assessment and family of normal distribution $N(\mu ,\sigma^{2})$ would be chosen for its distribution, the corresponding density could be written as:

(7) $$p\left(DI|M_{j}\right)=\int_{\sigma_{min}}^{\sigma_{max}}\int_{\mu_{min}}^{\mu_{max}}\frac{1}{\sigma \sqrt{2\pi }}e^{-\frac{{\left(DI-\mu \right)}^{2}}{2\sigma^{2}}}\pi_{j}\left(\mu ,\sigma \right)d\mu d\sigma ,$$

where $\mu_{min},\mu_{max},\sigma_{min},\sigma_{max}$ corresponds to lower and upper limits on mean value and standard deviation of Damage Index distribution respectively and $\pi_{j}$ is probability density on the parameter space (μ,σ).

### 3.2. Maximum Likelihood Method

The weights $w_{1},\ldots ,w_{k}$, the type of parametric families $p_{\left(\theta_{1},\ldots ,\theta_{n}\right)}\left(DI_{1},\ldots ,DI_{m}|M_{j}\right)$ determining DIs distribution and probability densities $\pi_{j}$ are the so-called a priori knowledge. The parameters $w_{1},\ldots ,w_{k}$ describing frequency of structural state occurrence is not related to approach to SHM and can be only estimated based on operational data or expert knowledge. However, the distribution $\pi_{j}$ on the parameter space can be inferred based on the training data, if there is no a priori knowledge available.

One of the methods for the definition of the families $p(DI_{1},\ldots ,DI_{m}|M_{j})$ based on training datasets for a given model $M_{j}$ is to use the Maximum Likelihood (ML) estimator and its asymptotic properties [53]. Given the training dataset $T_{j}$ for a structural state $M_{j}$, consisted of $N_{j}$ measurement outcomes represented in the form of the defined Damage Indices:

(8) $$T_{j}=\left\{\left(DI_{1,1},\ldots ,DI_{m,1}\right),\ldots ,\left(DI_{1},\ldots ,DI_{m,N_{j}}\right)\right\},$$

the so-called log-likelihood function *l* can be defined as:

(9) $$l\left(\theta_{1},\ldots ,\theta_{n}|M_{j}\right)=\sum_{i=1}^{N_{j}}\log\left(p_{\left(\theta_{1},\ldots ,\theta_{n}\right)}\left(DI_{1,i},\ldots ,DI_{m,i}|M_{j}\right)\right)$$

which is the logarithm of joint probability distribution measurements outcomes consisting of the training dataset. The ML estimators ${\hat{\theta }}_{1},\ldots ,{\hat{\theta }}_{n}$ for the unknown parameters $\left(\theta_{1},\ldots ,\theta_{n}\right)$ maximize the log-likelihood function, i.e.,:

(10) $$l\left({\hat{\theta }}_{1},\ldots ,{\hat{\theta }}_{n}|M_{j}\right)\geq l\left(\theta_{1},\ldots ,\theta_{n}|M_{j}\right),\;\left(\theta_{1},\ldots ,\theta_{n}\right)\in \Theta_{j}.$$

As the result of maximization procedure, family parameters ${\hat{\theta }}_{1},\ldots ,{\hat{\theta }}_{n}$ are selected, for which joint probability density of measurements outcomes consisting training dataset $T_{j}$ is the highest.

Maximum Likelihood algorithm not only selects the best parameters for representation of Damage Indices probability density $p_{\left(\theta_{1},\ldots ,\theta_{n}\right)}\left(DI_{1},\ldots ,DI_{m}|M_{j}\right)$ but it can also provide their measure of uncertainty, i.e., a priori distribution $\pi_{j}$ on the parameter space $\Theta_{j}$. For sufficiently large training datasets, it can be proven that the asymptotic distribution of maximum likelihood estimator can be approximated by *n*-dimensional normal distribution. In the context of Bayesian framework we propose to set the distribution $\pi_{j}$ on the parameter space as in Equation (6) as multivariate normal $\pi_{j}∼N\left({\hat{\theta }}_{1},\ldots ,{\hat{\theta }}_{n},\Sigma \right)$, where covariance matrix Σ can be estimated based on training dataset $T_{j}$ as well [53]. As admissible parameter space $\Theta_{j}$ can be assumed to be 95% joint confidence set for multivariate normal distribution $\pi_{j}∼N\left({\hat{\theta }}_{1},\ldots ,{\hat{\theta }}_{n},\Sigma \right)$. In general, it is an ellipsoid set based on Hotelling’s T-squared distribution ($T^{2}$) [68], but for simpler numerical implementation $\Theta_{j}$ is here proposed in the form of *n*-dimensional cuboid space $\Theta_{j}=\left[LCB_{1},UCB_{1}\right]\times \ldots \times \left[LCB_{n},UCB_{n}\right]$ where $LCB_{i}$, $UCB_{i}$ are the lower and the upper 95% intervals of one-dimensional normal marginal distribution for the *i*-th optimal parameter ${\hat{\theta }}_{i}$. Since marginal probability distribution for ${\hat{\theta }}_{i}$ can be approximated by $N({\hat{\theta }}_{i},\Sigma_{ii})$ where $\Sigma_{ii}$ is the *i*-th diagonal of the covariance matrix Σ, then:

(11) $$\begin{array}{cc} \; & LCB_{i}={\hat{\theta }}_{i}-1.96\sqrt{\Sigma_{ii}}, \end{array}$$

(12) $$\begin{array}{cc} \; & UCB_{i}={\hat{\theta }}_{i}+1.96\sqrt{\Sigma_{ii}}. \end{array}$$

Naturally, the proposed cuboid parameter subspace $\Theta_{j}$ is no longer the 95% confidence level set for the joint distribution $\pi_{j}∼N\left({\hat{\theta }}_{1},\ldots ,{\hat{\theta }}_{n},\Sigma \right)$ on the parameter space, but can be easier implemented numerically.

In the case of one single Damage Index DI and normal family of probability densities chosen for its distribution as in example given by Equation (7), ML estimators of the mean and the variance obtained for a training set $T_{j}=\left\{DI_{1},\ldots ,DI_{N_{j}}\right\}$ are [69]:

(13) $$\hat{\mu }=\frac{1}{N_{j}}\sum_{i=1}^{N_{j}}DI_{i}\equiv \bar{DI},\;\hat{\sigma }=\sqrt{\frac{1}{N_{j}}\sum_{i=1}^{N_{j}}{\left(DI_{i}-\bar{DI}\right)}^{2}}.$$

According to the assumptions, as covariance matrix Σ of the distribution $\pi_{j}$ we can set:

(14) $$\left(\begin{array}{cc} \frac{1}{N_{j}}{\hat{\sigma }}^{2}\equiv \Sigma_{11} & 0\\ 0 & \frac{2}{N_{j}}{\left({\hat{\sigma }}^{2}\right)}^{2}\equiv \Sigma_{22} \end{array}\right).$$

Then probability density $\pi_{j}\left(\mu ,\sigma \right)$ on parameter space in Equation (7) can be written in the following form:

(15) $$\pi_{j}\left(\mu ,\sigma \right)=\frac{1}{2\pi \sqrt{\Sigma_{11}\Sigma_{22}}}\exp\left(-\frac{{\left(\mu -\hat{\mu }\right)}^{2}}{2\Sigma_{11}}\right)\exp\left(-\frac{{\left(\sigma -\hat{\sigma }\right)}^{2}}{2\Sigma_{22}}\right)$$

and integration limits in Equation (7) can be adopted as in Equations (11) and (12).

There exist closed-form solutions for ML estimators ${\hat{\theta }}_{1},\ldots ,{\hat{\theta }}_{n}$ and covariance matrix Σ of their asymptotic distribution for popular families of probability densities [53] (mostly one-dimensional). Additionally, for one-dimensional ML problem, i.e., for single Damage Index used for structure assessment, there exist numerical tools for ML estimates determination of generic parametric probability families, including covariance matrix.

### 3.3. Principal Component Analysis Representation Space of Signal Features

For the purpose of practical implementation of ML algorithm, also numerical issues are important. Calculation of conditional densities (6) might be multidimensional if the Damage Indices $\left(DI_{1},\ldots ,DI_{m}\right)$ used for structure monitoring are correlated signal features. In particular, this can be the case for the approach presented in Section 2. The amplitude and phase of the forward transfer ratio can be correlated. Given a fixed structural state $M_{j}$, a series of measurements over range of frequencies could produce a set of data points which can be described by a tilted ellipse and a non-diagonal covariance matrix as in Figure 3. In that case, several parameters needed to describe measurement outcome distribution in parameter spaces $\Theta_{j}$ grows, since except of parameters required to determine marginal probabilities of a given signal feature, also their mutual dependencies, e.g., correlation coefficients, should be extracted from training data as well. Computational effort grows significantly with the dimensionality of the considered random variables. Application of multidimensional integration method from scipy.integrate Python package requires:

- 0.01 s for integration of 1-d normal distribution;
- 1.2 s for integration of 2-d normal distribution;
- 79.5 s for integration of 3-d normal distribution;

on a regular desktop PC.

In addition, closed-form solutions to ML optimization problem exist mostly for one-dimensional probability families, which is also the case of numerical ML optimization procedures since these are developed for univariate variables mostly. There exist few examples of packages for multidimensional covariates optimization in R language, e.g., mvnmle. However, these are designed for particular probability distributions (e.g., multivariate normal), and their numerical convergence might be poor. However, for one-dimensional random variables, there are implementations of ML method for a large family of probability distributions in most popular environments, e.g., MATLAB, R or Python. Moreover, some packages allow obtaining ML estimator for a generic parametrized random variable. In particular, in this paper class GenericLikelihoodModel of the module statsmodels implemented in Python environment was used for Bayesian classification based on ML algorithm implementation [70].

**Figure 4:**
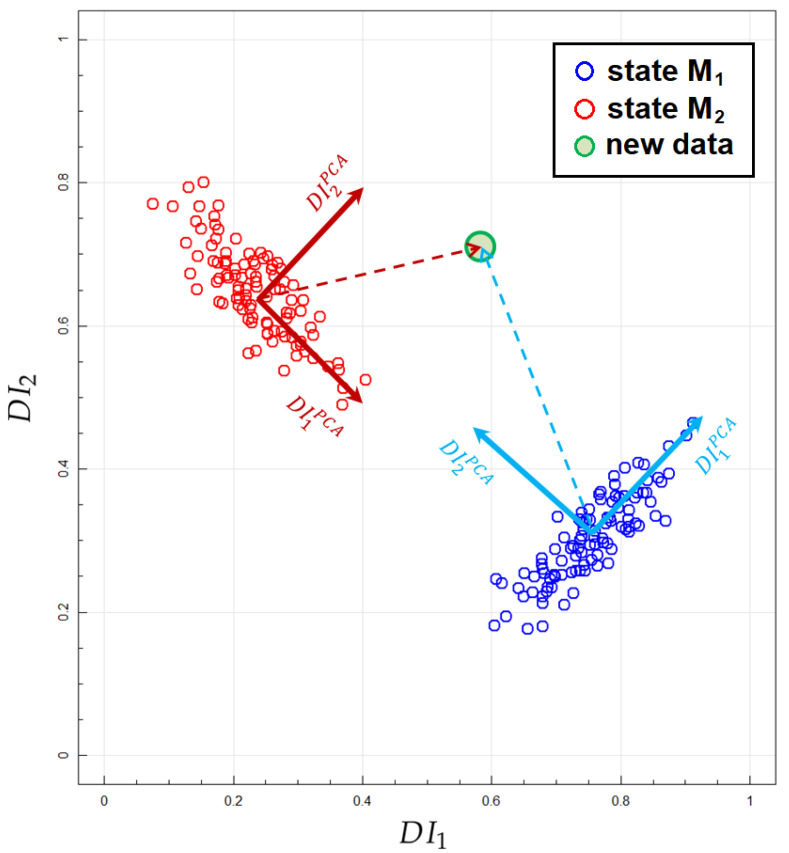
Example of the DIs in two dimensions obtained for two simulated structural states with indication of PCA transformed DIs spaces and vectors corresponding to new data point in both coordinate systems.

Therefore, in the implementation of the proposed algorithm for data classification based on ML estimators and Bayesian framework, decomposition of signal feature space in terms of Principal Components Analysis (PCA) [54] is applied. For training data set $T_{j}$ obtained for a structural state $M_{j}$, PCA allows for affine orthogonal transformation of the space $\left(DI_{1},\ldots ,DI_{m}\right)$ into new set of signal features $\left(DI_{1}^{j,PCA},\ldots ,DI_{m}^{j,PCA}\right)$, such that in terms of PCA derived features, the data in the training data set are uncorrelated. This is schematically presented in Figure 4 below. Then new measurement outcome in terms of the defined Damage Indices $\left(DI_{1},\ldots ,DI_{m}\right)$, which needs to be classified as corresponding to one of the structural states $M_{j}$, can be expressed in terms of PCA local coordinates systems derived for every training data set $T_{j}$.

In particular, every training data set:

(16) $$T_{j}=\left\{\left(DI_{1,1},\ldots ,DI_{m,1}\right),\ldots ,\left(DI_{1},\ldots ,DI_{m,N_{j}}\right)\right\},$$

of multivariate signal feature space can be transformed to training data set:

(17) $$T_{j}^{PCA}=\left\{\left(DI_{1,1}^{j,PCA},\ldots ,DI_{m,1}^{j,PCA}\right),\ldots ,\left(DI_{1}^{j,PCA},\ldots ,DI_{m,N_{j}}^{j,PCA}\right)\right\},$$

of the same dimensionality, but with signal features corresponding to subsequent Principal Components. In that way, no information is lost by the transformation; however, the transformed space of signal features is uncorrelated within the transformed training set due to the properties of PCA algorithm [54]. We propose to consider PCA transformed Damage Indices corresponding to different structural states $M_{j}$ as independent variables $\left(DI_{1}^{j,PCA},\ldots ,DI_{m}^{j,PCA}\right)$, so their multidimensional probability density $p(DI_{1}^{j,PCA},\ldots ,DI_{m}^{j,PCA}|M_{j})$ can be factorized as a product of one-dimensional probability densities $p(DI_{l}^{j,PCA})$, l=1,…,m. If $p(DI_{1}^{j,PCA},\ldots ,DI_{m}^{j,PCA}|M_{j})$ is assumed to be multivariate normal, then this property is satisfied without loss of generality. The next step is then to obtain one-dimensional ML estimators for every PCA derived feature $DI_{l}^{j,PCA}$, l=1,…,m separately. Under this assumption probability density in PCA transformed coordinates can be expressed as follows:

(18) $$p\left(DI_{1}^{j,PCA},\ldots ,DI_{m}^{j,PCA}|M_{j}\right)=\prod_{s=1}^{m}\int_{\Theta_{j,s}}p_{\left(\theta_{1},\ldots ,\theta_{l}\right)}\left(DI_{s}^{j,PCA}\right)\pi_{s}\left(\theta_{1},\ldots ,\theta_{l}\right)d\theta_{1}\ldots d\theta_{l},$$

where $p_{\left(\theta_{1},\ldots ,\theta_{l}\right)}$ are one-dimensional parametric probability families chosen to represent distribution of PCA transformed Damage Indices $p(DI_{l}^{j,PCA})$, l=1,…,m and $\pi_{s}∼N\left({\hat{\theta }}_{1,s},\ldots ,{\hat{\theta }}_{l,s},\Sigma^{s}\right)$, s=1,…,m are a priori multivariate normal probability densities on the parameter space $\left(\theta_{1},\ldots ,\theta_{l}\right)$ obtained from ML algorithm as described in the previous section. For the purpose of measurement outcome $\left(DI_{1},\ldots ,DI_{m}\right)$ classification corresponding to unknown state of the structure, it is first transformed to PCA derived coordinate systems for every defined structural states $M_{j}$ and then probability densities given by Equation (18) and a posteriori factors given by Equation (5) can be calculated.

Since there exist numerical procedures for obtaining ML estimates of generic one-dimensional probability families, the above procedure for data classification can be implemented practically. Yet Equation (18) involves in general multidimensional integration, which can significantly impact the performance as indicated in the beginning of this paragraph. Therefore the last step towards simplification of classification procedure is to disregard off-diagonal part of the covariance matrix Σ of ML-based a priori distributions $\pi_{s}∼N\left({\hat{\theta }}_{1,s},\ldots ,{\hat{\theta }}_{l,s},\Sigma^{s}\right)$, and assume that it is a product of one-dimensional normal distributions $N({\hat{\theta }}_{i,s},\Sigma_{ii}^{s})$, i=1,…,l:

(19) $$\pi_{s}\left(\theta_{1},\ldots ,\theta_{l}\right)=\frac{1}{\sqrt{2\pi \Sigma_{11}^{s}}}\exp\left(-\frac{{\left(\theta_{1}-{\hat{\theta }}_{1,s}\right)}^{2}}{2\Sigma_{11}}\right)\ldots \frac{1}{\sqrt{2\pi \Sigma_{ll}^{s}}}\exp\left(-\frac{{\left(\theta_{l}-{\hat{\theta }}_{l,s}\right)}^{2}}{2\Sigma_{ll}}\right),$$

where $\Sigma_{ii}$, i=1,…,l are diagonals of the estimated covariance matrix $\Sigma^{s}$ for ML estimators. For certain families of probabilities, e.g., as shown in Equation (14) the covariance matrix of ML estimator distribution is indeed diagonal.

### 3.4. Definition of Bayesian Setup for Voltage Transfer Ratio Approach to SHM

In the above sections the definition of Bayesian classifier based on ML estimators for general SHM approach was provided. In this section, its particular implementation, suited to be applied for the approach described in Section 2 is defined. In that case real and imaginary part of DIs defined in (3) can be considered to be suitable for Bayesian approach definition, i.e.,:

(20) $$DI_{1}=Re\left(DI\left(\omega \right)\right)=Re\left(\frac{FVTR(\omega )}{FVTR_{0}\left(\omega \right)}\right),DI_{2}=Im\left(DI\left(\omega \right)\right)=Im\left(\frac{FVTR(\omega )}{FVTR_{0}\left(\omega \right)}\right).$$

That is we consider $p(Re\left(DI\left(\omega \right)\right),Im\left(DI\left(\omega \right)\right)|M_{j})$ as a probability density on two-dimensional space which is not dependent on the excitation frequency ω within the defined frequency range. The details of measurement setup, in particular the adopted frequency range, are provided in Section 4. Therefore, we treat the result of measurement DI(ω)=(Re(DI(ω)),Im(DI(ω)) obtained for a given frequency as realization of random variable with probability distribution density $p(Re\left(DI\left(\omega \right)\right),Im\left(DI\left(\omega \right)\right)|M_{j})$ dependent on the state of the structure $M_{j}$. In particular, for the example presented in Figure 3 the Bayesian model could consist of two probability distributions from which DIs values in a given frequency range are drawn:

(21) $$p\left(Re\left(DI\left(\omega \right)\right),Im\left(DI\left(\omega \right)\right)|M_{1}\right),\;p\left(Re\left(DI\left(\omega \right)\right),Im\left(DI\left(\omega \right)\right)|M_{2}\right),$$

where $M_{1}$ corresponds to the pristine state of the structure and $M_{2}$ accounts for damage presence on a given sensing path.

For every defined structural state $M_{j}$ and corresponding training data set $T_{j}$ we perform PCA transformation of (Re(DI),Im(DI)) space as described in Section 3.3 and obtain local coordinate systems of signal features in which new data (Re(DI),Im(DI)) can be expressed for every $M_{j}$ (Figure 4). We assume that distribution of uncorrelated PCA transformed features belongs to one-dimensional normal family $N(\mu ,\sigma^{2})$ of distributions. Based on these assumptions and training data, we calculate ML estimators of μ and σ parameters for every PCA transformed feature and structural state $M_{j}$ as well as corresponding a priori distributions on the corresponding parameter space. For this purpose, we developed suitable software in Python environment with use of the class GenericLikelihoodModel of statsmodels module [70], but alternatively formulas given by Equations (13) and (14) could also be used. A priori distributions on the parameter space (μ,σ) in the form given by Equation (15) were adopted.

Measurement outcomes (Re(DI(ω)),Im(DI(ω))) which were supposed to be classified were expressed in terms of PCA determined signal features space for every structural state $M_{j}$. For every model probability density of the measurement outcome in PCA transformed spaces given by Equation (18) were calculated as well as corresponding a posteriori factors $PF_{j}$ as in Equation (5). The weights $w_{j}$ corresponding to structural states $M_{j}$ were set to be equal. Finally, the observed data (Re(DI(ω)),Im(DI(ω))) was classified to structural state $M_{j}$ for which the obtained a posteriori factor was the highest.

## 4. Experiment Results and Discussion

**Figure 5:**
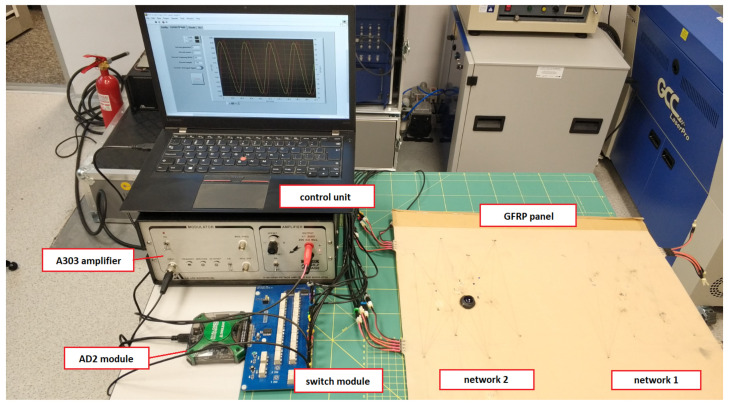
View of the acquisition system used in the experiment.

**Figure 6:**
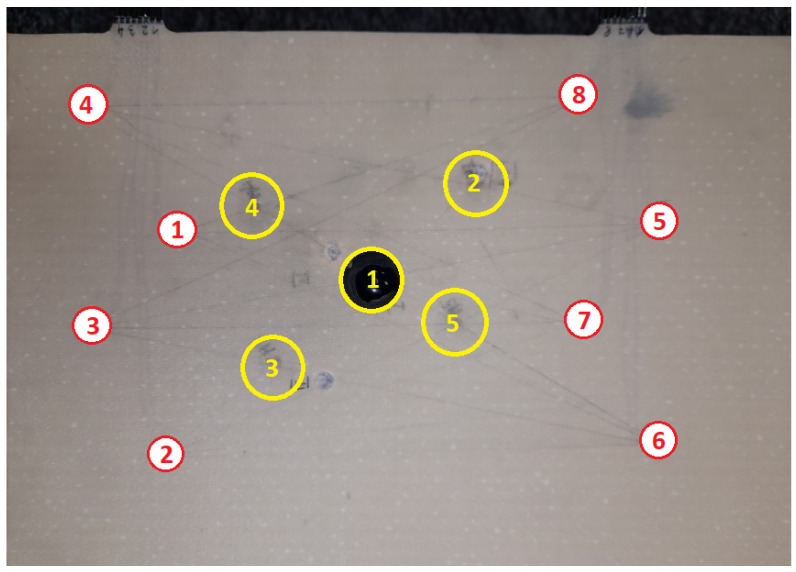
View of the selected specimen used in the experiment with indication of PZT sensors and artificial damage localization.

For the experiment, a GFRP composite panel equipped with two networks containing 8 PZT sensors each was used (Figure 5). Sensor localization was the same for both networks (Figure 6). Single layered PZT transducers produced by STEMINC (mod. SMD05T04R111WL), with a diameter equal to 5 mm, and thickness of 0.4 mm, made of SM111 material and of 450 ± 10 kHz resonant frequency [71] were used in the experiment. The sensors were embedded into the internal structure of the composite panel in its symmetry plane. For sensors excitation and signal acquisition, a dedicated system based on Analog Discovery 2 (AD2) module by DIGILENT [72] connected with eight channels relay switch module designed in the experiment hosting institution (ITWL) has been used (Figure 5). The signal generator was connected to A303 high voltage amplifier [73] to obtain a 100 Vpp (symmetric) harmonic excitation signal in the frequency range of 200–350 kHz.

### 4.1. Application of Voltage Transfer Ratio Approach to Artificial Damage Detection

Artificial damage was simulated for both networks by attaching a small mass to the surface of the panel at five predefined locations with the use of bitumen type paste (Figure 6). Excitation and response signals were collected for every pair of PZT transducers for the pristine state of the structure and with artificial damage introduced at predefined locations, which allowed for DIs calculation in accordance with Equation (3). The sensor used for elastic wave excitation was electrically isolated from the receiver, for measurement of the excitation $U_{in}$ as well as induced voltages $U_{out}$ standard oscilloscope probes were used. To diminish the noise, for a given frequency sinusoidal excitation was applied to PZT actuator and sinusoidal induced voltage was acquired on PZT receiver. Then the obtained input and output signals were averaged and FVTR coefficients were calculated on averaged signals. For every series of measurements and every sensing path of the network, only 50% of data closest to the point defined by median values of DIs obtained for the series were considered for analysis.

For both PZT networks, the following distinction of sensing paths with respect to introduced artificial damage has been introduced:

- Type I sensing paths which runs transversally through artificial damage and are sensitive to transmission mode of elastic waves interaction with damage;
- Type II sensing paths which are tangential to artificial damage or runs it its proximity and can be affected by transmission mode (to some extent) and reflection mode of wave interaction with damage;
- Type III sensing paths which are separated from artificial damage but are affected by waves reflected from damage;
- Type IV sensing paths which are well separated from damage and are not influenced by its presence.

**Figure 7:**
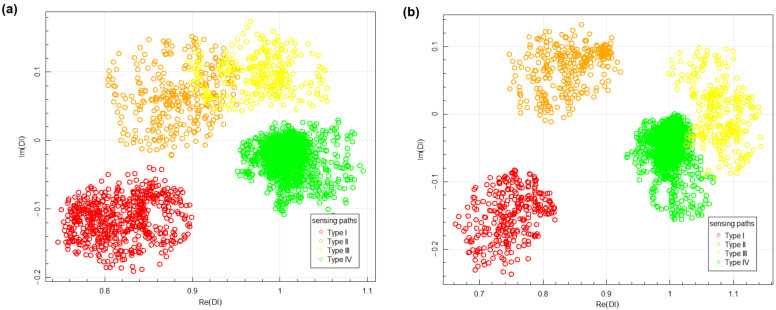
Examples of Damage Indices obtained for the first PZT network: (**a**) for damage in location no. 1; (**b**) for damage in location no. 3.

Therefore, in terms of Bayesian framework defined in Section 3, equivalents of four structural states were defined for every sensing path. In Figure 7 examples of Damage Indices obtained for two locations within the first PZT network, i.e., location no. 1 and location no. 3 (as shown in Figure 6), are presented. The following classification of sensing paths has been made in those examples:

- Type I sensing paths are defined by the following pair of PZT transducers:
  - for location no. 1: 3–5, 1–7, 2–8, 4–6;
  - for location no. 3: 2–8, 3–6.
- Type II sensing paths are defined by the following pair of PZT transducers:
  - for location no. 1: 1–6, 4–7;
  - for location no. 3: 3–7, 2–5.
- Type III sensing paths are defined by the following pair of PZT transducers:
  - for location no. 1: 3–7, 1–5;
  - for location no. 3: 2–7, 3–5.

The rest of the sensing paths of the network were not significantly influenced by damage presence, so these were classified as Type IV sensing paths. The rest of the data obtained for other artificial damage locations for both PZT networks were labeled in a similar manner.

Data obtained under seemingly identical conditions for undamaged state of the structure, i.e., Type IV sensing paths, can be used to assess the measurement noise. Absolute values of Type IV data obtained for damage location no. 1 (Figure 7a) are in the interval (0.95,1.10) and arguments are contained in $\left(-7^{\circ },2^{\circ }\right)$. The variability of forward voltage transfer ratio, i.e., FVTR as defined in Equation (1) is mainly due to noise of the voltage $U_{out}$ induced on PZT receiver, as being orders of magnitude smaller than the input voltage $U_{in}$. Additionally, since the voltage measurement is triggered on the raising slope of the input voltage $U_{in}$, it should not contribute to the phase variability of FVTR. Based on presented limiting values of the Damage Indices it can be assessed that the measurement noise in the amplitude of the output signals under seemingly identical conditions does not exceed 10% and variation of phase is less than $7^{\circ }$. If the dimensions of the network or attenuation properties of the medium are changed, these values could change as well since it could have significant impact on the output amplitudes.

Hotelling’s $T^{2}$ test was performed to assess statistical significance of difference between data corresponding to different type of sensing paths. The test was performed with use of Hotelling package in R environment [74]. The value of $T^{2}$ statistics obtained for data corresponding to damage location no. 1 between Type II and Type III sensing paths was 1038 with corresponding *p*-value equal to 0. Since these are groups of data least separated from each other (Figure 7a), this means very strong statistical significance between data obtained for different type of sensing paths under similar measurement conditions.

The results of measurement depend on relative location of damage and sensing path considered. The data corresponding to different types of sensing paths are in separated regions of the complex plane (Figure 7). This can be used for sensor location placement optimization algorithms [75] or modification of structure imaging algorithms, e.g., RAPID imaging algorithm [76,77,78] by introduction of additional sensing path geometrical mappings, depending on their type. Repeatability of indications was noticed for different sensing paths belonging to the same group. There are many factors which can impact results of structure assessment or working condition of monitored structure identification, e.g., in terms of loads [79]. Some of them, e.g., external measurement conditions such as temperature or stress level of the structure during measurement, can be either controlled or their influence can be diminished by proper definition of baseline signals data set, which can be acquired in broad variability range of such parameters. However, there are also hard to be controlled parameters which can also influence signals acquired by PZT sensors, e.g., strength of adhesive layer between sensor and monitored structure. Therefore, repeatability of a given Damage Index under similar structure conditions, but for different sensing paths is not always granted. In particular, proper normalization factors need to be used to take into account influence of length or the orientation of sensing path on values of Damage Index. In the presented study, Damage Indices obtained for different sensing paths but belonging to the same type of sensing paths influenced by damage, especially Type I and Type II are located in similar regions of the complex plane. In particular, Type I data are in the region contained in the domain (−0.65,0.9)×(−0.25,−0.05) of the complex plane for damage in location no. 1 and in location no. 3 (Figure 7). This result was obtained for sensing paths with different lengths and orientation for anisotropic GFRP medium, which indicates that Damage Index based on voltage transfer ratio (Equation (3)) allows for compensation of factors related to manufacturing of sensor network and anisotropies of monitored structure as well as anisotropies of piezoelectric properties of PZT sensors. This property is of significant importance for proper structure assessment based on the voltage transfer ratio approach to SHM, since it opens a possibility for proper SHM system calibration as well as application of data classification methods, Bayesian classifier in particular.

**Figure 8:**
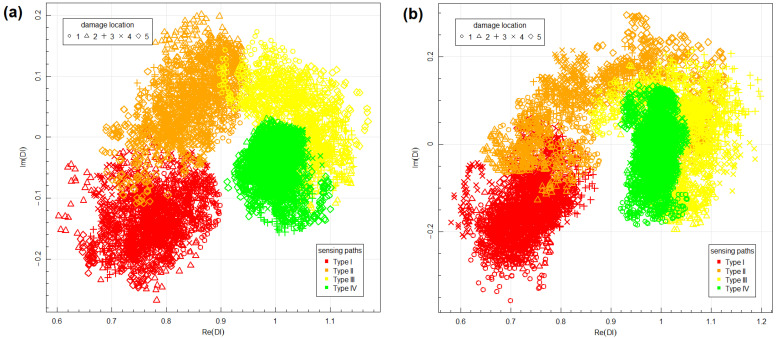
Aggregated Damage Indices obtained for different sensor networks: (**a**) network 1; (**b**) network 2.

Aggregated data for both networks are presented in Figure 8. The data corresponding to Type I and Type II sensing paths, which were sensitive on the transmission mode of elastic waves interaction with artificial damage, are well separated from DIs obtained for sensing paths not sensitive to damage (Type IV). For Type III sensing paths, the obtained data sets can intersect both with data acquired for sensing paths influenced by damage (Type II) as in location no. 1 (Figure 7a) as well as data corresponding to an undamaged state of the structure (Type IV) as in location no. 3 (Figure 7b), therefore it was observed that transmission mode of elastic wave interaction with damage has a stronger effect on DIs than reflection mode of interaction. Additionally, it can be noticed that the variance of data is higher for Type II and Type III sensing paths, which are sensitive to the reflection mode of wave interaction with damage. This can be related to the influence of damage presence on phase change of received signal for such sensing paths. Phase change can depend on damage-respective localization of damage and sensing path, which can cause higher variation of data in those groups.

Based on the obtained data, the efficiency and properties of the proposed Bayesian classifier have been studied. For that purpose, the bootstrap data resampling method was applied [80] in accordance with the following procedure implemented using boot library available in the R environment. Data acquired for network one corresponding to different groups and obtained for randomly selected sensing path and measurement series were used for Bayesian model definition. Then training data set was removed from the bootstrap sample, and 100 data points of the remaining set were randomly selected and classified using the obtained model for model validation, both using the data obtained for network one and network two. The percentage of correct and misclassified results was determined, and the next step of the bootstrap resampling procedure was initiated. Similarly, the nearest-neighbor classifier performance was verified. For this algorithm, the majority class of 20 nearest neighbors to a given data point was used as a basis for classification. In the Table 1, Table 2, Table 3 and Table 4 below, confusion matrices of Bayesian and nearest-neighbor classifiers are presented, which were calculated based on mean values of classification rates obtained for different groups of data after 300 steps of the bootstrap procedure in both cases.

**Figure 9:**
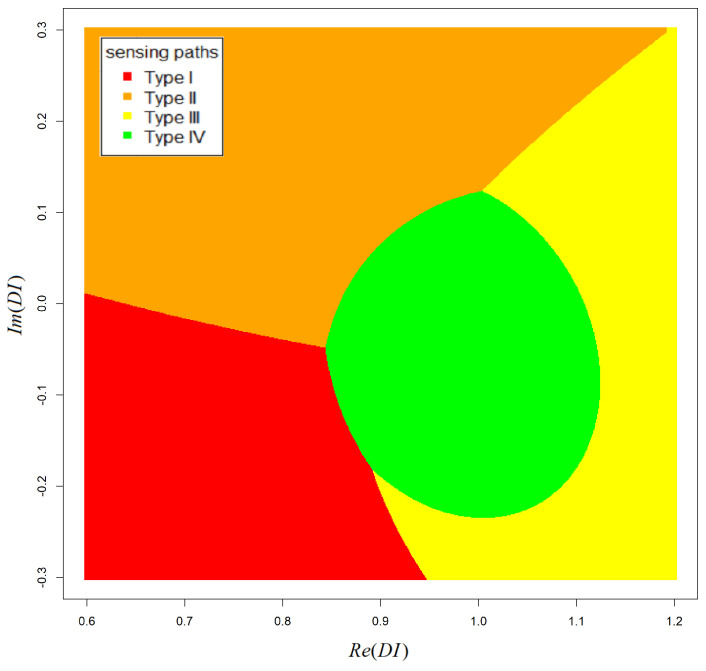
Example of Bayesian classifier decision boundaries obtained for experimental data.

It can be noticed that the Bayesian classifier is characterized by a very low percentage of false-positive indications and reduced sensitivity to small disturbances of signal due to damage (which is the case for Type III sensing paths). For sensing paths not influenced by damage (Type IV), the probability of DIs misclassification is less than 3%, and the probability of detection of Type III data is 2–23%. As mentioned above, for sensing paths not sensitive to the reflection mode of wave interaction with damage, data variance is smaller. Therefore, ML estimators used in the proposed method tend to be more accurate for those groups of data, which after integration over hyperparameter space (6) results in more compact classification domains than for Type II and Type III groups of data (Figure 9). In those regions, a posteriori probability of class correspondence is higher for Type IV data, even if a significant number of other types of data are contained there, which is not the case for the nearest-neighbor model. A reduced ratio of false-positive indications can be of significant importance for particular applications, e.g., in aerospace, where costs of unplanned maintenance procedures can be very high. In such cases, reduction of false calls ratio can be critical to the practical application of the system.

### 4.2. Application of Voltage Transfer Ratio Approach to Impact Damage Detection

**Figure 10:**
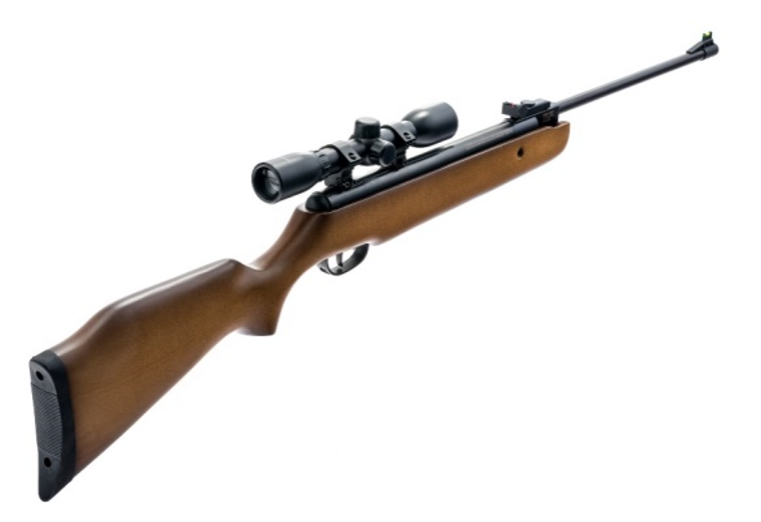
Pellet gun used to introduce impact damage.

**Figure 11:**
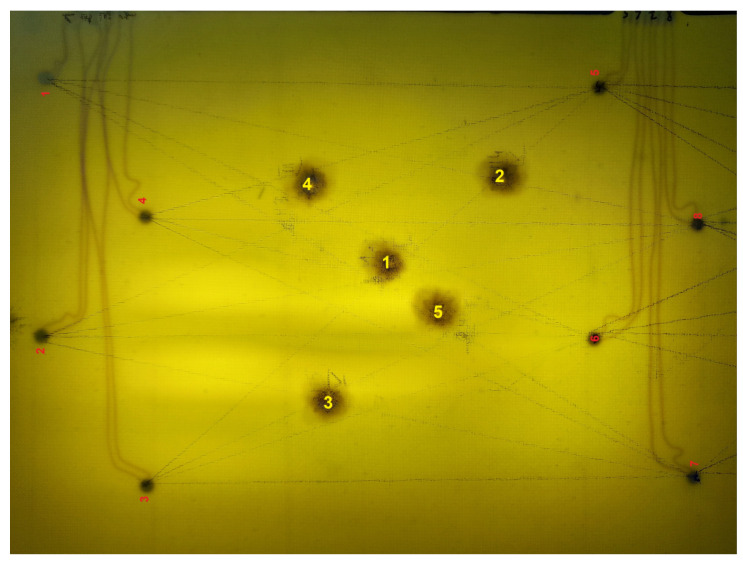
Barely Visible Impact Damage introduced in the composite panel (network 2).

For additional, practical verification of the method, damage caused by impacts of low energies were introduced within network no. 2. For that purpose, an air gun able to provide not more than 17 J of kinetic energy to the pellet with an initial speed not higher than 300 m/s was used (Figure 10). The specimen was subjected to five shoots which caused Barely Visible Impact Damage (BVID) in the proximity of artificial damage locations (Figure 11).

Measurements were conducted for the pristine state of the structure and after each subsequent impact. In the case of BVID damage, the data were divided into three groups:

- Type I sensing paths which runs transversally through BVID;
- Type II sensing paths which runs in close proximity of impact damage;
- Type III sensing paths which are separated from impact damage and are barely influenced by its presence.

**Figure 12:**
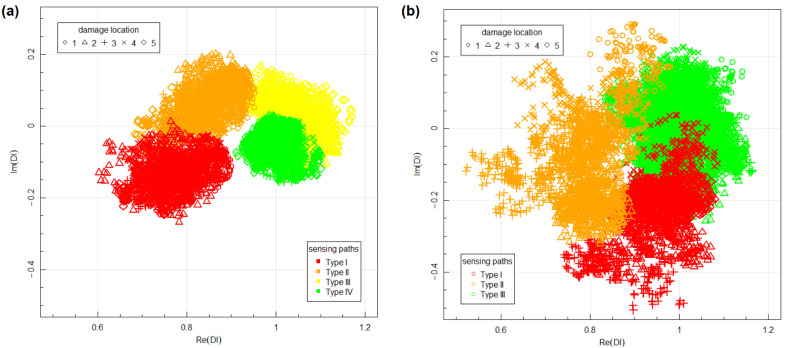
Aggregated Damage Indices obtained for different type of damage: (**a**) artificial damage (network 1); (**b**) BVID damage (network 2).

The DIs obtained for BVID damage on network two are presented in Figure 12. Impact damage affects acquired signals more significantly than artificial damage, resulting in a higher variance of data corresponding to different groups. Therefore, differentiation between sensing paths running in the proximity of damage to two groups, as in the case of artificial damage experiment, was not possible. Data obtained for Type II sensing paths for BVID damage are in region of complex plane similar to Type I and Type II data obtained for artificial damage and data for Type III sensing paths least influenced by BVID damage (Figure 12b) covers the domain occupied by data corresponding to Type III and Type IV sensing paths in the case of artificial damage. As indicated in Figure 1 BVID damage can introduce severe change of local mechanical properties of the structure, therefore its impact on signal acquired by PZT receiver can be more significant.

Signals acquired for network 1 in the presence of artificial damage were used as training data for Bayesian and nearest-neighbor model definition and data classification in the presence of BVID damage within network 2. The confusion matrix for Bayesian classifier is provided in Table 5 and in Table 6 for nearest-neighbor classifier.

Bayesian classifier trained on artificial damage resulted in a very low false-positive ratio for BVID damage—4% compared to 16% of false calls obtained for the nearest-neighbor classifier. Signals acquired for sensing paths running in the proximity of BVID damage but not intersecting them were more heavily influenced by structural damage than by artificial mass. A significant fraction of data for Type II sensing paths, i.e., 78% for Bayesian and 54% for the nearest-neighbor classifier, were classified as Type I sensing paths. Additionally, in the case of Type I sensing paths, more significant shift in the phase of received signals and spread of data were observed (Figure 12) which caused a significant fraction of those data being classified as undamaged type (Type IV), i.e., 34% for Bayesian and 29% for nearest-neighbor classifier.

## 5. Summary

In this paper, an approach to SHM based on sinusoidal excitation of PZT transducers and voltage transfer ratio-based Damage Indices has been proposed. The approach was applied to artificial and BVID damage detection of composite structures. Based on the proposed signal features it was possible to detect both type of damage. Additionally, it was possible to differentiate data obtained for sensing paths that are sensitive to the transmission mode of elastic waves interaction with damage and those capturing waves reflected from it. In principle, the method can be applied to detect all types of damage which can influence the amplitude or the phase of output voltage induced on elastic waves receiver. It is expected it could be applied to detection of fatigue cracks or corrosion damage of metallic components as well as impact damage or debonding of composite structures. However, further studies are required to confirm damage detection capabilities in those scenarios.

Importantly, the method proved to be repeatable under similar measurement conditions, i.e., damage type and its relative position and orientation with respect to sensing paths. Measurements were performed for anisotropic medium using two independent networks of PZT transducers and independently introduced damage in different specimen locations. This property is of significant importance for proper structure assessment based on the voltage transfer ratio approach to SHM, since it opens the possibility of proper SHM system calibration on test structures and transfer the obtained results to target components. It was demonstrated in the paper that the method is robust enough to apply data classification with damage detection rate above 90% and with acceptable ratio of false-positive indications below 3% (Table 1 and Table 3). In the authors opinion such performance should be acceptable for SHM systems on early stage of development, especially with respect to false-positive indications as such events generate additional maintenance cost due to unnecessary non-destructive inspections. It is worth noting that such a result was obtained for classifiers trained on data acquired for the PZT network independent from the network for which they were applied for signals classification, as would be the case when the SHM method is used in practice.

In addition to voltage transfer ratio approach to SHM, a new Bayesian approach to SHM data classification was proposed in the paper. The definition of the classifier is general, it can be applied to SHM systems based on Damage Indices approach to structure assessment, not necessarily obtained with the use of voltage transfer ratio approach described in the paper, although the efficiency and validity of the proposed classifier in other approaches is an open issue. The classifier definition is based on asymptotic properties of ML method and Principal Component Analysis for orthogonal data transformation; the only a priori data necessary for its definition is the type of one-dimensional probability families underlying distribution of SHM data for a single class and weights corresponding to a different type of data. The efficiency and validity of the classifier were confirmed based on the experimental data. Its damage detection rate was comparable with a well-known nearest-neighbor classifier, i.e., above 93% for Bayesian classifier and above 99% for nearest-neighbor method while maintaining a significantly lower false calls ratio, i.e., below 3% versus below 20% respectively (Table 1, Table 2, Table 3 and Table 4).

Further work can be focused on verification of voltage transfer ratio approach in detection of other damage types than considered in this article, e.g., fatigue cracks or corrosion of metallic components. Another open question is to study vulnerability of the proposed Damage Indices to influence of external measurement conditions, e.g., the temperature or load level of the monitored structure, as such parameters can influence the amplitude and the phase of voltage induced on PZT transducers. Additionally, properties of the proposed Bayesian classifier should be further studied, in particular its efficiency and validity when applied to other SHM systems based on Damage Indices approach to structure assessment.

## Figures and Tables

**Table 1:** Rate of classification of Bayesian model for network 1.

		True Class			
		Type I	Type II	Type III	Type IV
**result class**	Type I	0.93	0.04	0	0
	Type II	0.02	0.89	0.11	0
	Type III	0.05	0	0.02	0
	Type IV	0	0.07	0.87	1

**Table 2:** Rate of classification of nearest-neighbor model for network 1.

		True Class			
		Type I	Type II	Type III	Type IV
**result class**	Type I	0.99	0.12	0.00	0
	Type II	0.01	0.87	0.19	0
	Type III	0	0	0.26	0.20
	Type IV	0	0.01	0.55	0.80

**Table 3:** Rate of classification of Bayesian model for network 2.

		True Class			
		Type I	Type II	Type III	Type IV
**result class**	Type I	0.97	0.17	0	0
	Type II	0.03	0.70	0.12	0
	Type III	0	0.01	0.23	0.03
	Type IV	0	0.12	0.65	0.97

**Table 4:** Rate of classification of nearest-neighbor model for network 2.

		True Class			
		Type I	Type II	Type III	Type IV
**result class**	Type I	0.99	0.24	0	0
	Type II	0.01	0.58	0.20	0
	Type III	0	0.04	0.33	0.18
	Type IV	0	0.14	0.47	0.82

**Table 5:** Rate of classification of Bayesian classifier for BVID data (network 2) based on training data obtained for artificial damage (network 1).

		True Class		
		Type I	Type II	Type III
**result class**	Type I	0.66	0.78	0
	Type II	0	0.06	0
	Type III	0	0.11	0.04
	Type IV	0.34	0.05	0.96

**Table 6:** Rate of classification of nearest-neighbor classifier for BVID data (network 2) based on training data obtained for artificial damage (network 1).

		True Class		
		Type I	Type II	Type III
**result class**	Type I	0.71	0.54	0
	Type II	0	0.42	0.15
	Type III	0	0.04	0.01
	Type IV	0.29	0	0.84

## Data Availability

Data used in this study are available on-demand from the corresponding author.

## Abbreviations

The following abbreviations are used in this manuscript:

BVID	Barely Visible Impact Damage
DI (DIs)	Damage Index (Damage Indices)
EMI	Electromechanical Impedance
GFRP	Glass Fiber Reinforced Polymer
ML	Maximum Likelihood
PCA	Principal Component Analysis
SHM	Structural Health Monitoring

## References

[1] Fraden J. (2010). Handbook of Modern Sensors.

[2] Boller C., Chang F.K., Fujino Y. (2009). Encyclopedia of Structural Health Monitoring.

[3] Staszewski W., Boller C., Tomlinson G. (2004). Health Monitoring of Aerospace Structures.

[4] Mukhopadhyay S.C. (2011). New Developments in Sensing Technology for Structural Health Monitoring.

[5] Adams D. (2007). Health Monitoring of Structural Materials and Components: Methods with Applications.

[6] Heywang W., Lubitz K., Wersing W. (2008). Piezoelectricity: Evolution and Future of a Technology.

[7] Giurgiutiu V. (2014). Structural Health Monitoring: With Piezoelectric Wafer Active Sensors.

[8] Su Z., Ye L. (2009). Identification of Damage Using Lamb Waves: From Fundamentals to Applications.

[9] Stepinski T., Uhl T., Staszewski W. (2013). Advanced Structural Damage Detection: From Theory to Engineering Applications.

[10] Yang J. (2005). An Introduction to the Theory of Piezoelectricity.

[11] Mei H., Haider M.F., Joseph R., Migot A., Giurgiutiu V. (2019). Recent advances in piezoelectric wafer active sensors for structural health monitoring applications. Sensors.

[12] Dragan K., Dziendzikowski M. (2016). A method to compensate non-damage-related influences on Damage Indices used for pitch-catch scheme of piezoelectric transducer based Structural Health Monitoring. Struct. Health Monit..

[13] Janapati V., Kopsaftopoulos F., Li F., Lee S.J., Chang F.K. (2016). Damage detection sensitivity characterization of acousto-ultrasound-based structural health monitoring techniques. Struct. Health Monit..

[14] Yadav S.K., Mishra S., Kopsaftopoulos F., Chang F.K. (2021). Reliability of crack quantification via acousto-ultrasound active-sensing structural health monitoring using surface-mounted PZT actuators/sensors. Struct. Health Monit..

[15] Amerini F., Meo M. (2011). Structural health monitoring of bolted joints using linear and nonlinear acoustic/ultrasound methods. Struct. Health Monit..

[16] Rucka M. (2018). Monitoring steel bolted joints during a monotonic tensile test using linear and nonlinear Lamb wave methods: A feasibility study. Metals.

[17] Li N., Wang F., Song G. (2020). Monitoring of bolt looseness using piezoelectric transducers: Three-dimensional numerical modeling with experimental verification. J. Intell. Mater. Syst. Struct..

[18] Demetgul M., Senyurek V.Y., Uyandik R., Tansel I., Yazicioglu O. (2015). Evaluation of the health of riveted joints with active and passive structural health monitoring techniques. Measurement.

[19] Mickens T., Schulz M., Sundaresan M., Ghoshal A., Naser A., Reichmeider R. (2003). Structural health monitoring of an aircraft joint. Mech. Syst. Signal Process..

[20] Li W., Liu T., Gao S., Luo M., Wang J., Wu J. (2019). An electromechanical impedance-instrumented corrosion-measuring probe. J. Intell. Mater. Syst. Struct..

[21] Li W., Liu T., Zou D., Wang J., Yi T.H. (2019). PZT based smart corrosion coupon using electromechanical impedance. Mech. Syst. Signal Process..

[22] Dai W., Wang X., Zhang M., Zhang W., Wang R. (2019). Corrosion monitoring method of porous aluminum alloy plate hole edges based on piezoelectric sensors. Sensors.

[23] Lim Y.Y., Kwong K.Z., Liew W.Y.H., Soh C.K. (2016). Non-destructive concrete strength evaluation using smart piezoelectric transducer—A comparative study. Smart Mater. Struct..

[24] Lim Y.Y., Smith S.T., Padilla R.V., Soh C.K. (2021). Monitoring of concrete curing using the electromechanical impedance technique: Review and path forward. Struct. Health Monit..

[25] Zhang J., Zhang C., Xiao J., Jiang J. (2019). A PZT-based electromechanical impedance method for monitoring the soil freeze–thaw process. Sensors.

[26] Zhang C., Wang X., Yan Q., Vipulanandan C., Song G. (2020). A novel method to monitor soft soil strength development in artificial ground freezing projects based on electromechanical impedance technique: Theoretical modeling and experimental validation. J. Intell. Mater. Syst. Struct..

[27] Tua P., Quek S., Wang Q. (2005). Detection of cracks in cylindrical pipes and plates using piezo-actuated Lamb waves. Smart Mater. Struct..

[28] Yan S., Li Y., Zhang S., Song G., Zhao P. (2018). Pipeline damage detection using piezoceramic transducers: Numerical analyses with experimental validation. Sensors.

[29] Song H., Lim H.J., Sohn H. (2013). Electromechanical impedance measurement from large structures using a dual piezoelectric transducer. J. Sound Vib..

[30] Abbas M., Shafiee M. (2018). Structural health monitoring (SHM) and determination of surface defects in large metallic structures using ultrasonic guided waves. Sensors.

[31] Hameed M.S., Li Z., Chen J., Qi J. (2019). Lamb-wave-based multistage damage detection method using an active PZT sensor network for large structures. Sensors.

[32] Marzani A., Testoni N., De Marchi L., Messina M., Monaco E., Apicella A. (2020). An open database for benchmarking guided waves structural health monitoring algorithms on a composite full-scale outer wing demonstrator. Struct. Health Monit..

[33] Menegaz G.L., Tsuruta K.M., Finzi Neto R.M., Steffen V., Araujo C.A., Guimarães G. (2021). Use of the electromechanical impedance method in the detection of inclusions: Application to mammary tumors. Struct. Health Monit..

[34] Junior P., D’addona D.M., Aguiar P.R., Teti R. (2018). Dressing tool condition monitoring through impedance-based sensors: Part 1—PZT diaphragm transducer response and EMI sensing technique. Sensors.

[35] Junior P., D’Addona D.M., Aguiar P.R., Teti R. (2018). Dressing tool condition monitoring through impedance-based sensors: Part 2—Neural networks and k-nearest neighbor classifier approach. Sensors.

[36] Roach D.P., Neidigk S. (2011). Does the Maturity of Structural Health Monitoring Technology Match User Readiness.

[37] Giurgiutiu V. (2015). Structural Health Monitoring of Aerospace Composites.

[38] Hale J. (2006). 787 From The Ground Up. Aero Mag..

[39] Gay D. (2015). Composite Materials: Design and Applications.

[40] Bielawski R. (2017). Composite materials in military aviation and selected problems with implementation. Rev. Air Force Acad..

[41] Talreja R. (2003). Fatigue of composite materials. Modern Trends in Composite Laminates Mechanics.

[42] Poon C., Benak T., Gould R. (1990). Assessment of impact damage in toughened resin composites. Theor. Appl. Fract. Mech..

[43] Li C., Hu N., Yin Y., Sekine H., Fukunaga H. (2002). Low-velocity impact-induced damage of continuous fiber-reinforced composite laminates. Part I. An FEM numerical model. Compos. Part A Appl. Sci. Manuf..

[44] Bieniaś J., Jakubczak P., Surowska B., Dragan K. (2015). Low-energy impact behaviour and damage characterization of carbon fibre reinforced polymer and aluminium hybrid laminates. Arch. Civ. Mech. Eng..

[45] Tie Y., Zhang Q., Hou Y., Li C. (2020). Impact damage assessment in orthotropic CFRP laminates using nonlinear Lamb wave: Experimental and numerical investigations. Compos. Struct..

[46] Smith R., Jones L., Zeqiri B., Hodnett M. (1998). Ultrasonic C-scan standardisation for fibre-reinforced polymer composites: Minimising the uncertainties in attenuation measurements. Insight.

[47] Tuloup C., Harizi W., Aboura Z., Meyer Y., Khellil K., Lachat R. (2019). On the use of in-situ piezoelectric sensors for the manufacturing and structural health monitoring of polymer-matrix composites: A literature review. Compos. Struct..

[48] Diamanti K., Hodgkinson J.M., Soutis C. (2004). Detection of Low-velocity Impact Damage in Composite Plates using Lamb Waves. Struct. Health Monit..

[49] Ochôa P., Infante V., Silva J.M., Groves R.M. (2015). Detection of multiple low-energy impact damage in composite plates using Lamb wave techniques. Compos. Part B Eng..

[50] Dziendzikowski M., Kurnyta A., Dragan K., Klysz S., Leski A. (2016). In situ Barely Visible Impact Damage detection and localization for composite structures using surface mounted and embedded PZT transducers: A comparative study. Mech. Syst. Signal Process..

[51] De Luca A., Caputo F., Khodaei Z.S., Aliabadi M. (2018). Damage characterization of composite plates under low velocity impact using ultrasonic guided waves. Compos. Part B Eng..

[52] Dziendzikowski M., Niedbala P., Kurnyta A., Kowalczyk K., Dragan K. (2018). Structural health monitoring of a composite panel based on PZT sensors and a transfer impedance framework. Sensors.

[53] Taboga M., Maximum likelihood In Lectures on Probability Theory and Mathematical Statistics, 3rd ed.; Kindle Direct Publishing: 2017; Volume Online appendix. https://www.statlect.com/fundamentals-of-statistics/maximum-likelihood.

[54] Hastie T., Tibshirani R., Friedman J. (2009). The Elements of Statistical Learning: Data Mining, Inference, and Prediction.

[55] Mori N., Biwa S., Hayashi T. (2013). Reflection and transmission of Lamb waves at an imperfect joint of plates. J. Appl. Phys..

[56] Annamdas V.G., Radhika M.A. (2013). Electromechanical impedance of piezoelectric transducers for monitoring metallic and non-metallic structures: A review of wired, wireless and energy-harvesting methods. J. Intell. Mater. Syst. Struct..

[57] Na W.S., Baek J. (2018). A review of the piezoelectric electromechanical impedance based structural health monitoring technique for engineering structures. Sensors.

[58] Lee J.W. (2021). An Experimental Study on Bolt Looseness Monitoring Using Low-Cost Transfer Impedance Technique. Int. J. Steel Struct..

[59] Chiu W.K., Koh Y., Galea S.C., Rajic N. (2000). Smart structure application in bonded repairs. Compos. Struct..

[60] Bhalla S., Gupta A., Bansal S., Garg T. (2009). Ultra low-cost adaptations of electro-mechanical impedance technique for structural health monitoring. J. Intell. Mater. Syst. Struct..

[61] Tenreiro A.F.G., Lopes A.M., da Silva L.F. (2021). A review of structural health monitoring of bonded structures using electromechanical impedance spectroscopy. Struct. Health Monit..

[62] Flynn E.B., Todd M.D. (2010). A Bayesian approach to optimal sensor placement for structural health monitoring with application to active sensing. Mech. Syst. Signal Process..

[63] Rogers T., Worden K., Fuentes R., Dervilis N., Tygesen U., Cross E. (2019). A Bayesian non-parametric clustering approach for semi-supervised structural health monitoring. Mech. Syst. Signal Process..

[64] Huo H., He J., Guan X. (2020). A Bayesian fusion method for composite damage identification using Lamb wave. Struct. Health Monit..

[65] Zhang Y.M., Wang H., Wan H.P., Mao J.X., Xu Y.C. (2020). Anomaly detection of structural health monitoring data using the maximum likelihood estimation-based Bayesian dynamic linear model. Struct. Health Monit..

[66] Yang Y., Chadha M., Hu Z., Vega M.A., Parno M.D., Todd M.D. (2021). A probabilistic optimal sensor design approach for structural health monitoring using risk-weighted f-divergence. Mech. Syst. Signal Process..

[67] Taboga M., Bayesian inference In Lectures on Probability Theory and Mathematical Statistics, 3rd ed.; Kindle Direct Publishing: 2017; Volume Online appendix. https://www.statlect.com/fundamentals-of-statistics/Bayesian-inference.

[68] Mujica L., Rodellar J., Fernandez A., Güemes A. (2011). Q-statistic and T2-statistic PCA-based measures for damage assessment in structures. Struct. Health Monit..

[69] Taboga M., Normal distribution—Maximum Likelihood Estimation In Lectures on Probability Theory and Mathematical Statistics, 3rd ed.; Kindle Direct Publishing: 2017; Volume Online appendix. https://www.statlect.com/fundamentals-of-statistics/normal-distribution-maximum-likelihood.

[70] Statsmodels. https://www.statsmodels.org/stable/examples/notebooks/generated/generic_mle.html.

[71] Steminc. https://www.steminc.com/PZT/en/piezo-disc-transducer-450-khz.

[72] Digilent. https://store.digilentinc.com.

[73] A.A. Lab Systems Ltd.. https://www.lab-systems.com/products/amplifier/a303.html.

[74] R Project. https://cran.r-project.org/.

[75] Yang C., Liang K., Zhang X., Geng X. (2019). Sensor placement algorithm for structural health monitoring with redundancy elimination model based on sub-clustering strategy. Mech. Syst. Signal Process..

[76] Liu Z., Zhong X., Dong T., He C., Wu B. (2017). Delamination detection in composite plates by synthesizing time-reversed Lamb waves and a modified damage imaging algorithm based on RAPID. Struct. Control Health Monit..

[77] Dziendzikowski M., Dragan K., Katunin A. (2017). Localizing impact damage of composite structures with modified RAPID algorithm and non-circular PZT arrays. Arch. Civ. Mech. Eng..

[78] Wang S., Wu W., Shen Y., Liu Y., Jiang S. (2020). Influence of the PZT sensor array configuration on Lamb wave tomography imaging with the RAPID algorithm for hole and crack detection. Sensors.

[79] Yang C. (2021). A novel uncertainty-oriented regularization method for load identification. Mech. Syst. Signal Process..

[80] Shao J., Tu D. (2012). The Jackknife and Bootstrap.

